# Unlocking the Tumor Microenvironment: Innovations in Multiplex Immunohistochemistry

**DOI:** 10.3390/cells14221819

**Published:** 2025-11-20

**Authors:** Bipin Gupta, George Yang, Marc Key

**Affiliations:** 1 Diagnostic Biosystems, Pleasanton, CA 94588, USA; george.yang@dbiosys.com; 2 Key Biomedical Services, Ojai, CA 93023, USA; marc@keybiomedical.com

**Keywords:** tumor microenvironment, immune cells, inflammation, immunohistochemistry, multiplex staining

## Abstract

The immune control of cancer growth is an area of active investigation. In this study, we demonstrated the feasibility of using standard immunohistochemistry methods in conjunction with a set of newly developed chromogens to demonstrate immune cell markers in a multiplex staining system. Immune infiltrating cells in breast cancer were identified using antibodies to CD20 (B-cells), CD3 (T-cells), and CD163 (macrophages). Additionally, the tumor compartment was identified using cytokeratin (AE1/AE3), and Ki67 was used to determine the proliferation index. These stains showed a significant immune cell infiltrate surrounding and within the tumors. B-cells, T-cells, and macrophages were abundant at the tumor periphery, particularly in areas where tertiary lymphoid structures were also present. In contrast, B-cells were significantly reduced within the tumor interior compared to an abundant infiltrate of T-cells and macrophages. Patterns of B-cell, T-cell, and macrophage infiltration were identified. Depending upon the particular set of markers chosen for analysis, a simple visual examination, without the aid of computer-assisted imaging systems, was sufficient to differentiate up to five different markers.

## 1. Introduction

The interactions between immune cells and other components of the tumor microenvironment collectively promote tumor growth, metastasis, and responses to therapies. However, the complexity of this microenvironment presents a significant challenge in exploring these components and understanding their interactions. In light of these challenges, the tumor microenvironment has become an area of active investigation into cancer biology and the interplay between tumor cells and their surrounding environment. The tumor microenvironment provides the essential requirements for tumor progression. It comprises tumor cells, immune cells, supporting stromal cells, and endothelial cells, as well as an extracellular matrix, cytokines, and other bioactive molecules. Major cellular components of the immune system include T-cells, B-cells, tumor-associated macrophages, and natural killer cells. The role of immune cells in both promoting and suppressing tumor growth has been extensively reviewed [1,2].

It is now well established that significant cellular heterogeneity exists in a wide range of tumors. Despite extensive efforts to analyze cellular infiltrates, single-cell assays, such as nucleic acid sequencing and flow cytometry [3,4,5,6], do not provide crucial spatial information, thus highlighting the urgent need for improved techniques.

Pathologists may describe some spatial information, such as the magnitude and distribution of inflammatory cells, during histological evaluation of tissue specimens stained with hematoxylin and eosin (H&E). The abundance of inflammatory responses can be described using terms such as “minimal, moderate, or severe.” In particular, the evaluation of tumor-infiltrating lymphocytes (TILs) has gained support as a valuable biomarker due to their association with prolonged survival and response to treatment [7,8,9,10].

These studies have prompted several investigations into the role of lymphocytic infiltrates in solid tumors [10,11]. To evaluate TILs in H&E tissue sections of breast cancer, the TIL Working Group of the International Immuno-Oncology Biomarkers Working Group has published guidelines for scoring the percentage of TILs [12]. These guidelines, which are considered a significant advancement in the field, include a microscopic assessment of TILs in H&E tissue sections of breast cancer to identify tumor-associated stroma, the type and magnitude of the inflammatory infiltrate, and to report the percentage of the tumor-associated stroma containing immune cell infiltrates [13].

Despite advances in deciphering the role that immune cells play in controlling breast cancer, there remains a lack of reproducible methods to assess immune infiltration based solely on H&E histological sections. Several studies have demonstrated that automated image analysis can be used to accurately assess and score heterogeneous cell types in breast cancer based on the analysis of H&E sections [13,14,15]. In particular, using this approach, studies have shown that specific lymphocyte populations among all cell types present were predictive of disease-specific survival in breast cancer, including triple-negative tumors, HER2/neu-positive tumors, and hormone receptor-positive tumors, thus demonstrating the efficacy and accuracy of image analysis in identifying immune cells in H&E-stained tissues [16,17,18]. The value of image analysis combined with machine learning to characterize lymphocytic infiltrates and their spatial distribution in breast cancer patients has now been confirmed by several investigations [13,14,15,16,17,18].

In addition to conventional H&E stains, immunohistochemistry (IHC) provides an alternative tool for analyzing immune cell infiltrates. Furthermore, IHC has the advantage of classifying the type and origin of the cellular infiltrates. Using cell-type-specific antibodies, it is possible to classify immune infiltrates according to their specific cell type, such as B-cell, T-cell, and macrophage. However, IHC is not routinely used to identify and classify subsets of immune cells in breast cancer. This may be partially due to time constraints, desire to preserve valuable diagnostic tissues, or absence of robust methods to combine multiple stains within a single slide. In instances where spatial analysis of IHC images has been performed, most studies have focused on morphological patterns [18]. Meanwhile, a reliable metric of spatial heterogeneity at the single-cell level has proven elusive.

This investigation highlights the efficacy of multiplex staining for visualizing immune cell infiltration into breast cancers. We demonstrate that standard immunohistochemical methods can be used for identifying the major subsets of immune cells, including B-cells, T-cells, and macrophages. This method should provide another tool for extracting morphological, spatial, and immunological information from highly multiplex-stained images.

## 2. Materials and Methods

### 2.1. Sample Preparation

Human tissue specimens were obtained from surgical samples as paraffin blocks. Tissues were fixed in formalin and embedded in paraffin (FFPE) using standard histological methods. Tissue blocks were sectioned at 4 µm, adhered to positively charged microscope slides, and stored at room temperature until the time of staining. In preparation for staining, the tissue sections were deparaffinized through a series of graded solutions of xylene and alcohol and then rehydrated in deionized water. Following deparaffinization, the tissues were firmly attached to the microscope slides using an adherence-promoting reagent (Tissue Glue, Diagnostic Biosystems). Deparaffinized tissue sections were then subjected to antigen retrieval by submerging slides in a solution of Tris-EDTA, pH 9.0 and heating in a pressure cooker at 15 psi for 15 min.

### 2.2. Materials

Antibodies were initially screened by IHC on tonsil tissues as single stains to determine the optimal dilutions. All antibodies were obtained from Diagnostic Biosystems, Pleasanton, CA, USA and are listed in Table 1.

Staining methods, reagents, and chromogens used in these studies have been previously described [19]. Table 2 lists all chromogens used for multiplex staining. All chromogens were obtained from Diagnostic Biosystems.

### 2.3. Immunohistochemistry

Immunohistochemical staining was performed using a sequential multiplex staining method due to its flexibility for using any combination of antibodies and enzyme systems. The methods for multiplex immunohistochemistry have been previously described [19]. Four representative breast adenocarcinomas were randomly selected for a detailed analysis of host immune cell infiltration.

Ki67 was evaluated by estimating the percentage of stained tumor cells using HRP-Green as the fifth color in a multiplex stain. For this analysis, a global distribution was used, and a score was calculated by visual analysis of representative high-power fields. The estimated percentage was rounded to the nearest quartile (<1, 25, 50, 75 and 100 percent). For this analysis, Ki67 staining of immune cells was disregarded.

### 2.4. Microscopic Evaluation

Stained slides were viewed under brightfield microscopy at 100× and 400× magnification. Slides were evaluated for specific and background staining and graded on a scale of 0 to 3 in 0.5 grade increments, with 0 indicating no staining, 1 indicating light staining, 2 indicating moderate staining, and 3 indicating intense staining.

## 3. Results

**Figure 1:**
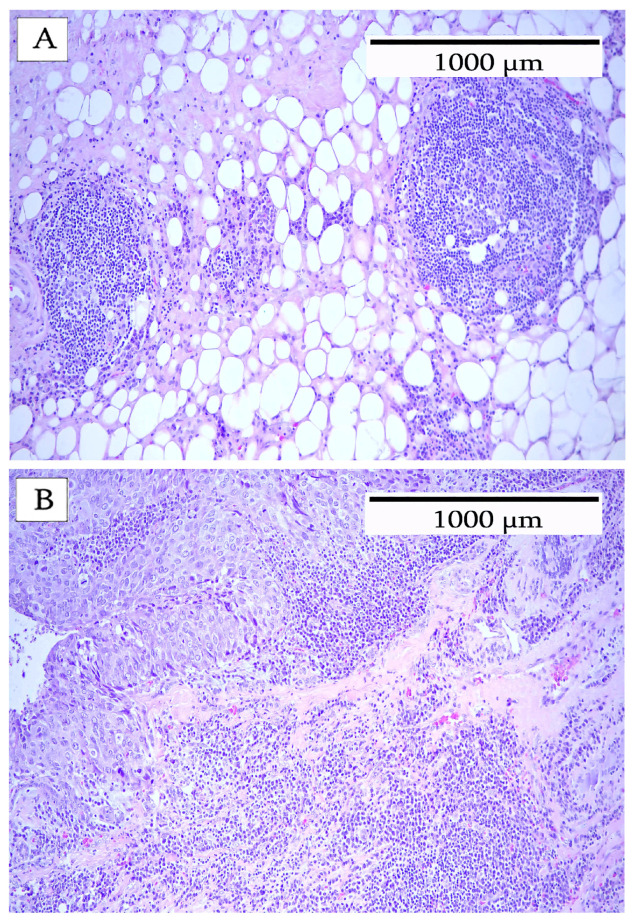
Hematoxylin and eosin stain of breast carcinoma. Area at the tumor periphery with a lymphocytic infiltrate. (**A**) shows a TLS with a clearly defined follicular structure. (**B**) shows an undefined lymphocytic infiltrate lacking a follicular structure.

Breast adenocarcinomas were stained with hematoxylin and eosin (H&E) for morphological examination of the host cell infiltrate. An example of an H&E image is shown in Figure 1. The low-power image revealed a significant influx of inflammatory immune cells and tertiary lymphoid structures (TLSs) characterized by germinal centers and follicular structures. Other inflammatory cells were observed in abundance in both the intratumoral and the peripheral locations of the carcinoma. This particular image shows a well-defined TLS that was easily identified in the H&E image.

**Figure 2:**
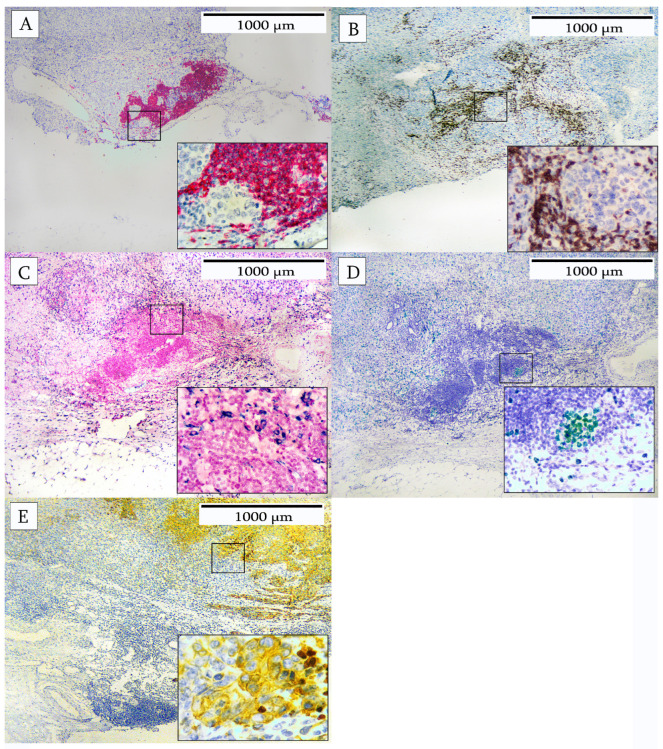
Tumor-infiltrating lymphocytes in breast carcinoma. (**A**) identifies the predominant cell type as a CD20+ B-cell (red). (**B**) shows numerous CD3+ T-cells (brown) at the periphery of the TILs. (**C**) shows CD163-positive macrophages (blue-black) surrounding the TILs. (**D**) confirms that the TILs contain actively proliferating B-cells, as identified by Ki67-positive cells (green). (**E**) shows the location of the cytokeratin-positive tumor cells (yellow) in relation to the TILs.

**Figure 3:**
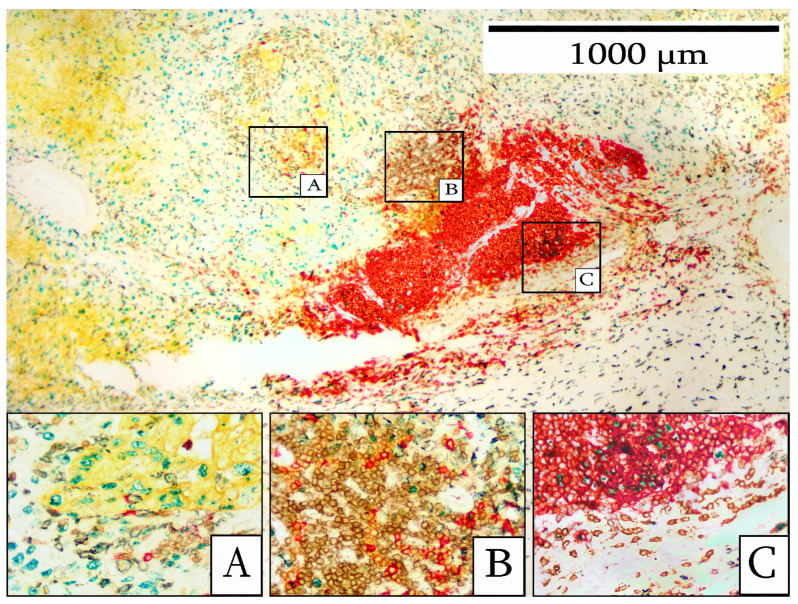
Immune cells surrounding an area of tumor-infiltrating lymphocytes. Box (**A**) shows cytokeratin-positive tumor cells (yellow) in relation to the TILs. Box (**B**) shows an area composed primarily of CD3+ T-cells (brown). A few scattered B-cells (red) are present. Box (**C**) shows a B-cell area with a central region of Ki67-positive cells (green). Occasional CD163-positive macrophages (blue-black) are visible in this region. The large boxes (**A**–**C**) are the high-power views corresponding to the smaller boxes A, B, and C.

**Figure 4:**
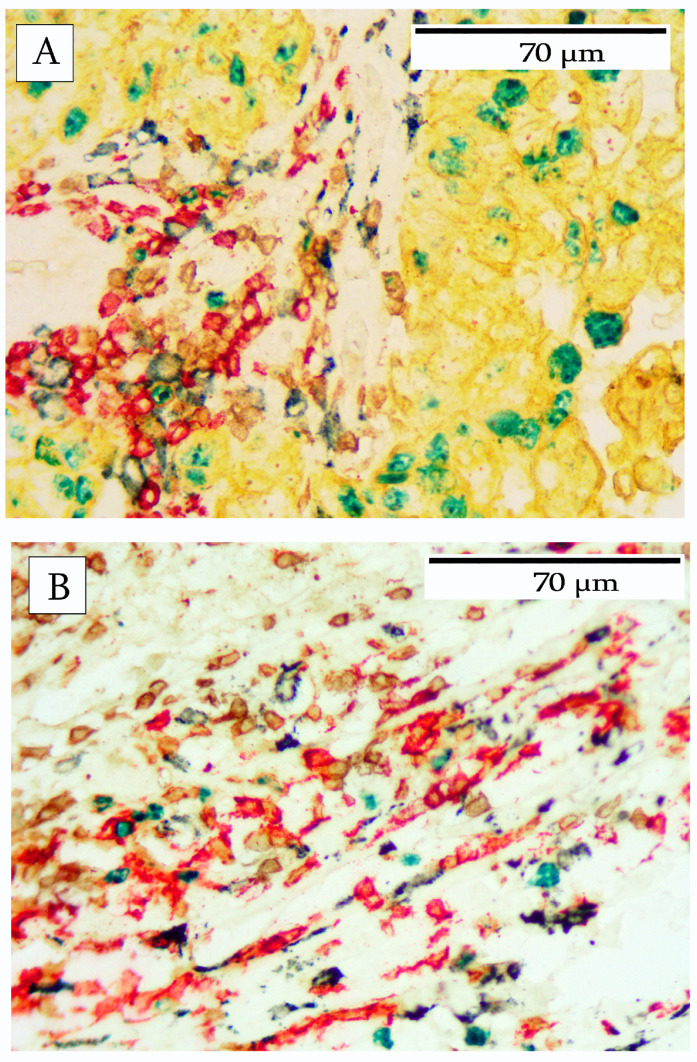
Immune cell infiltrate in breast carcinoma. (**A**) depicts the intratumoral area, depicting cytokeratin-positive tumor cells (yellow) with a high Ki67 index (green). CD20 B-cells (red), CD3 T-cells (brown), and CD163 macrophages were present. (**B**) shows the peritumoral stroma surrounding the tumor. All three types of immune cells were present, including CD20 B-cells (red), CD3 T-cells (brown), and CD163 macrophages (blue-black). Occasional Ki67-positive immune cells (green) were observed.

**Figure 5:**
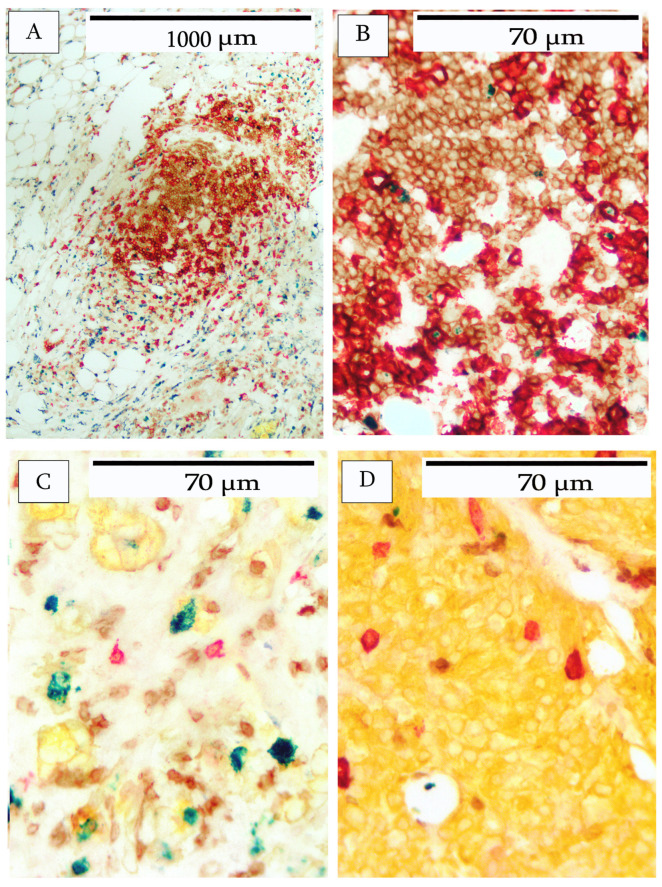
Patterns of lymphocyte infiltration in breast carcinoma. (**A**) shows an area of lymphocyte aggregations but lacking a follicular structure. (**B**) shows a higher magnification of the lymphocyte aggregate showing that it is composed of both CD20 B-cells (red) and CD3 T-cells (brown). (**C**) depicts occasional CD20 B-cells (red) and more abundant CD3 T-cells (brown) within tumor nests. Tumor cells were identified by AE1/AE3 cytokeratin (yellow). Ki67-positive cells (green) were observed. (**D**) shows a tumor area (yellow) containing a few large infiltrating CD20+ cells (red).

**Figure 6:**
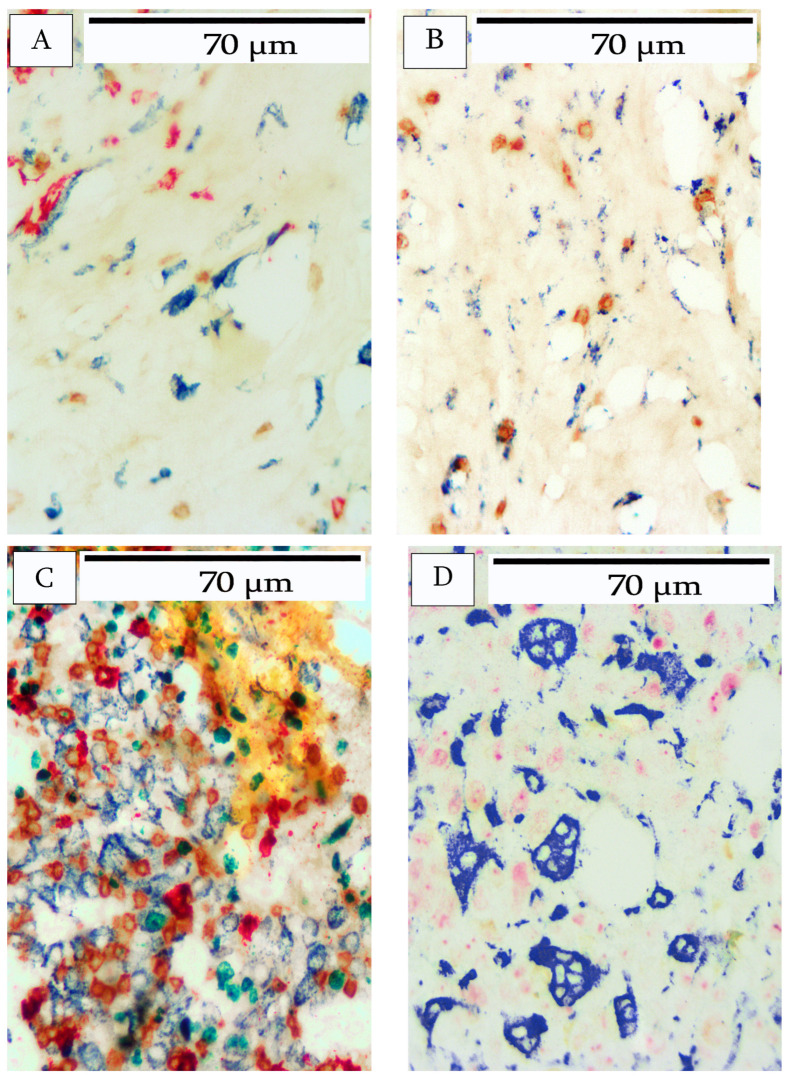
Patterns of macrophage infiltration into breast carcinoma. (**A**) shows a typical macrophage infiltrate. Macrophages with a spindle-shaped morphology (blue) were abundant in the stroma. (**B**) depicts macrophages with a spindle-shaped morphology (blue) but fragmented. (**C**) depicts macrophages with an epithelioid morphology surrounded by a heavy infiltrate of both B-cells (red) and T-cells (brown). (**D**) shows a necrotic area of the tumor containing macrophages with large phagocytic vacuoles. The adjacent areas show the remnants of CD20+ cells.

In another breast carcinoma, analysis was performed by multiplex IHC to identify the cellular composition of the immune cell infiltrate. The cells were identified as CD20 B-cells, CD3 T-cells, and CD163 macrophages. The tumor compartment was identified using cytokeratin and proliferating cells as indicated by Ki67. Various patterns of immune cell infiltrates are depicted in Figure 2, Figure 3, Figure 4, Figure 5 and Figure 6. Figure 2 illustrates an area characterized predominantly by tumor-infiltrating lymphocytes (TILs). The individual immunohistochemical stains are shown in Figure 2A–E and reveal a large number of CD20+ B-cells, CD3+ T-cells, and CD163-positive macrophages. Ki67-positive cells were present in a B-cell area of the infiltrate. Tumor cells were stained with cytokeratin to indicate their location with respect to the TILs.

Figure 3 illustrates the multiplex stain for the five-antibody panel which identifies the various cell types present. Tumor cells frequently showed positive staining for Ki67. The TILs exhibited a discernible organizational structure, with a predominance of CD20+ B-cells at the center and CD3+ T-cells at the periphery. CD163+ macrophages were observed surrounding the TILs. Ki67-positive B-cells were present. By IHC staining, it was apparent that this lymphocytic infiltrate shared certain features in common with a more mature TLS, including a predominantly B-cell population with a central area of Ki67-positive cells and a peripheral area of T-cells.

Figure 4 illustrates a typical staining pattern of infiltrating immune cells, which are present in both intratumoral and peritumoral locations. Figure 4A shows the intratumoral infiltrating immune cells, including CD20 B-cells, CD3-T-cells, and CD163 macrophages. Highly activated immune cells, as indicated by Ki67 labeling, were also observed in the intratumoral area. Additionally, the carcinoma cells were stained with cytokeratin and Ki67.

Figure 4B demonstrates the peritumoral stroma with heavy infiltrates of lymphocytes comprising CD20 B-cells, CD3 T-cells, and CD163 macrophages. Occasional immune cells were positive for Ki67.

Table 3 summarizes the general characteristics of each tumor including morphological description and Ki67 indices.

The immune cell infiltrate was characterized using antibodies for B-cells (CD20), T-cells (CD3), and macrophages (CD163). Each of these cell types was characterized in terms of its morphology, abundance, and location. These results are summarized in Table 4.

The immune cell infiltrates demonstrated various organizational arrangements within and between different tumors. Despite the abundant cellular infiltrates and the complexity of the images, specific patterns were clearly discernible and are shown in Figure 5 and Figure 6.

In addition to the heavy lymphocyte infiltrate with some organizational structure (see Figure 2), there were more loosely organized lymphoid clusters, comprising B-cells and T-cells. Ki67-positive lymphocytes were either absent or present at very low numbers (Figure 5A,B). In intratumoral areas, B-cells invaded tumor nests primarily as single cells, and were less abundant than T-cells (Figure 5C). In one tumor, a large CD20+ cell was present in very low numbers within the tumor nests (Figure 5D). This particular tumor had a very low Ki67 index (<1%).

Similarly, macrophages, as identified by CD163, exhibited a range of various morphological shapes and patterns. Typically, macrophages were abundant in peritumoral areas within connective tissue and adipose tissue. Macrophages were also present in lower numbers within the tumor stroma. Macrophages within stromal areas typically presented with a spindle shape (Figure 6A) and occasionally as fragmented cells (Figure 6B). Within the tumor nests, and particularly in areas of heavy infiltration, the macrophages exhibited a more epithelioid appearance (Figure 6C). In one tumor with significant necrosis, the macrophages appeared as large vacuolated cells, presumably phagocytic cells (Figure 6D). Another aspect of this particular pattern was occasional B-cells, but no T-cells. Furthermore, CD20+ debris was abundant and the faint outlines of the remnants of presumptive B-cells were present.

## 4. Discussion

Several studies have demonstrated that TILs can serve as a biomarker to predict clinical outcomes, including treatment response, in patients with invasive breast cancer [20,21,22,23,24,25,26,27]. The role of tumor-associated macrophages is less clear. Tumor-associated macrophages have typically been associated with a poor prognosis and drug resistance; however, some studies suggest that macrophage activation can have a tumor-suppressing effect [28]. With the emergence of new immunotherapies for the treatment of breast cancer, the evaluation of tumor-infiltrating immune cells is of increased importance. However, the routine evaluation of immune cell infiltrates has not gained widespread adoption despite the relatively straightforward methodology proposed by the International Immuno-Oncology Biomarker Working Group. This methodology was based on the examination of H&E-stained tissue sections and utilized tools and methods already in standard practice [12].

Studies using computer-aided image analysis to identify lymphocytic infiltrates in H&E-stained images has provided another important tool for more precise quantitation of lymphocytic infiltrates. However, these methods cannot classify lymphocyte subsets based on their immunophenotype. Compared to T-cells, B-cells employ fundamentally different mechanisms for distinguishing the self from non-self [29]. Such mechanisms may also be active in tumor surveillance. Accordingly, across diverse cancer types, B-cells have provided greater prognostic value than T-cells [30,31].

Fluorescence-based IHC offers another technique to highlight cells and structures, and it can also be used to evaluate TILs and other types of immune cells. Furthermore, immunofluorescence can classify subsets of lymphocytes within the broader class of TILs. The Opal™ multiplex staining system, useful for histological sections, can stain up to nine dfferent fluorchromes based on tyramide chemistry [32]. Such high-density multiplex stains require special imaging devices such as those provided by multispectral imaging systems capable of capturing images at different wavelengths and performs the separation and quantification of multiple fluorescent signals [33,34].

In practice, many diagnostic laboratories lack access to such systems, yet they are still capable of providing standard IHC testing. The multiplex staining method presented in this study can be easily integrated into any pathology laboratory that is proficient in IHC staining but may lack access to digital pathology platforms. Although brightfield IHC is not routinely used for this purpose, with improved IHC staining methods, pathologists will be able to add the evaluation of TILs and other immune cells to the standard IHC panel of ER, PR, and HER2/neu expression.

To facilitate the standardization and reproducibility of classifying immune cell infiltrates, objective IHC methods are urgently needed. Such approaches must take into account the complexity of immune infiltration, including identification of lymphocyte subsets as well as macrophages. H&E-stained slides are inadequate for evaluating this complexity. To address this issue, we have developed a new approach based on classical IHC chromogenic methods that can be easily incorporated into most pathology laboratories without modification of existing equipment or practices [19]. The method relies on various chromogenic substrates that provide excellent color separation when viewed by brightfield microscopy. Sequential IHC achieves multiplex staining with antibody elution performed between each sequential step. With this approach, the feasibility of identifying up to five different markers on a single microscope slide with excellent color differentiation has been demonstrated. Furthermore, the time required to achieve these results was within a few hours, making this method feasible within the typical laboratory’s daily workflow.

Depending on the specific antibody panel chosen for multiplex staining, the complexity of the information obtained can be overwhelming, making it challenging for the microscopist to extract all relevant information solely by visual interpretation. Such situations can benefit from computer-aided image analysis using suitable algorithms [35,36,37,38,39]. In the present study, to reduce complexity, we primarily focused on identifying the three major categories of immune infiltrating cells: B-cells, T-cells, and macrophages. In addition we have identified the tumor component and the proliferating fraction of the tumor cell component. These components were easily recognizable visually without the aid of image analysis. Different patterns of immune cell infiltration were observed. In particular, various patterns of lymphocyte aggregations were recognized. Lymphoid aggregates frequently develop at sites of chronic infection, autoimmune disease, allograft rejection, and tumors [40,41,42,43]. Recent studies suggest that TLS in tumors may reflect a localized immune response with endogenous production of anti-tumor antibodies and is associated with a favorable prognosis and response to immunotherapy [35]. The evaluation of immune infiltrates in invasive breast cancer has become increasingly important, serving as a key biomarker that can predict clinical outcomes and treatment responses. The expanded use of this biomarker is likely to expand in the future with the emergence of new treatment modalities.

## 5. Conclusions

This study examines the spatial heterogeneity of immune cell infiltration in breast cancer using immunohistochemical techniques. Utilizing newly developed chromogens in conjunction with standard staining methods, this research identifies immune cells, including B-cells, T-cells, and macrophages, in samples of breast tumors. Notably, B-cells were concentrated at the tumor periphery, while T-cells and macrophages were prominent within the tumor interior. This investigation highlights the efficacy of multiplex staining in visualizing these cell types and contributes to our understanding of the tumor microenvironment. Additionally, it addresses the limitations of conventional H&E staining, and emphasizes the need for more robust methods of immunohistochemistry for a more accurate assessment of immune cell distribution.

## Figures and Tables

**Table 1:** Antibody list for multiplex staining.

Part					
Antibody	Species	Clone	Number	Dilution	Multiplex Stain
CD3	Mouse	LN10	Mob474	1:50	Brown (DAB)
CD20	Mouse	L26	Mob004	1:80	Red (AP-Red)
CD163	Mouse	10D6	Mob460	1:50	Blue (HRP-Blue)
Ki67	Rabbit	SP6	RMAB004	1:80	Green (HRP-Green)
Cytokeratin	Mouse	AE1/AE3	Mob190	1:40	Yellow (HRP-Yellow)

**Table 2:** List of chromogens.

Chromogen	Part Number	Enzyme System
PermaBlue-HRP	K063	Peroxidase
PermaGreen-HRP	K074	Peroxidase
PermaYellow-HRP	K060	Peroxidase
Stable DAB	K047	Peroxidase
PermaRed-AP	K049	Alkaline Phosphatase

**Table 3:** Breast cancer morphology and Ki67 index.

Breast Cancer	Morphology	Immune Cell Infiltrate	Ki67 Index
1	Moderately differentiated adenocarcinoma	Moderate	5%
2	Moderately differentiated adenocarcinoma	Heavy	75%
3	Moderately differentiated adenocarcinoma	Heavy	50%
4	Moderately differentiated adenocarcinoma	Minimal	<1%

**Table 4:** Description of immune cell infiltrate.

Breast Cancer	B-Cells	T-cells	Macrophages
1	Predominantly peripheral location. Occasional lymphoid aggregates lacking follicular structure.	Uniformly distributed. Infiltration into tumor nests.	Slight infiltration. Predominantly peripheral location in stroma and adipose tissue.
2	Infiltration throughout tumor. Tertiary lymphoid structures comprise B- and T-cells and occasional Ki67-positive B-cells at the periphery.	Heavy infiltration throughout the tumor.	Slight infiltration. Predominantly peripheral location in stroma and adipose tissue.
3	Tertiary lymphoid structures at the periphery comprise B- and T-cells. Occasional Ki67-positive B-cells. Heavy B-cell infiltrate throughout tumor.	Heavy T-cell infiltrate throughout tumor.	Moderate macrophage infiltration in the periphery of the stroma and adipose tissue. Slight infiltrate into interior tumor stroma.
4	Peripheral location and within stromal areas of the tumor. Occasional large CD20+ cells within tumor nests.	Peripheral location and within stromal areas of tumor.	Peripheral location and within stromal areas of tumor.

## Data Availability

The original contributions presented in this study are included in the article. Further inquiries can be directed to the corresponding author.
